# COVID-19 and Obstetrical Care: Coping With New Stress

**DOI:** 10.7759/cureus.12116

**Published:** 2020-12-16

**Authors:** Ritu Sharma, Shikha Seth, Hariom K Solanki, Neha Mishra, Anurag Srivastava, Kiran Jakhar

**Affiliations:** 1 Department of Obstetrics and Gynaecology, Government Institute of Medical Sciences, Greater Noida, IND; 2 Department of Community Medicine, Government Institute of Medical Sciences, Greater Noida, IND; 3 Department of Obstetrics and Gynaecology, Goverment Institute of Medical Sciences, Greater Noida, IND; 4 Department of Obstetrics and Gynaecology, All India Institute of Medical Sciences, New Delhi, IND; 5 Department of Psychiatry, Government Institute of Medical Sciences, Greater Noida, IND

**Keywords:** mental health outcomes, covid 19 pandemic, stressors, coping strategies, obstetrical care

## Abstract

Objective

Our study aimed to assess the mental health outcomes and coping strategies among healthcare workers (HCWs) in an already over-burdened maternity ward and labour room during the coronavirus disease 2019 (COVID-19) pandemic.

Methods

This cross-sectional questionnaire survey was conducted using Google Forms (Google LLC, Mountain View, CA), which included demographic characteristics, perceived stressors, and validated scales: the Depression, Anxiety and Stress Scale - 21 Items (DASS-21), Insomnia Severity Index, and the Brief Coping Orientation to Problems Experienced (Brief COPE) scale. The results were evaluated and compared among COVID-19 caregivers and other HCWs.

Results

A total of 184 participants were included in the study, out of which 112 (60.9%) were COVID-19 caregivers. Overall, HCWs managing COVID-19 patients experienced significantly higher levels of depression, anxiety, and stress. They often adopted an avoidant coping style (p-value: 0.006). The results of binary logistic regression analysis revealed that living with family and perceiving multiple stressors appeared to be associated with increased risk of anxiety while being a COVID-19 caregiver and appeared to be a risk factor for stress. Avoidant coping was found to be associated with insomnia while approach coping was less associated with anxiety.

The most prevalent stressor among HCWs at our institute was distancing from family and friends (62%) followed by fear of getting infected (51.1%). Compared to other HCWs, the stressors perceived in significantly higher proportion by COVID-19 caregivers included distancing from family and friends (p-value: 0.003), scarcity of workforce (p-value: 0.005), and dealing with non-cooperative patients (p-value: <0.001).

Conclusion

We would request the immediate attention of the concerned authorities to implement interventions to buffer the impact of COVID-19 in the already stressed-out maternity wards and labour rooms.

## Introduction

The World Health Organization (WHO) declared the coronavirus disease 2019 (COVID-19) outbreak a pandemic on March 11, 2020, and there has been a significant rise in infections among both the general public and healthcare workers (HCWs) since then [1-3]. The specialties like obstetrics and gynaecology are managing COVID-19 pregnancies by only limited evidence-based protocols [4]. In general, outpatient care has been put on hold by other specialties, but such an approach is not feasible in obstetrics. The already understaffed maternity wards are facing additional problems such as a rapidly changing environment, unsafe workplace, and rapidly changing employee shift patterns [5-8]. With the increasing number of COVID-19 pregnancies, HCWs in maternity wards and labour rooms continue to take additional risks in order to manage the crisis. The objective of our study is to evaluate mental health outcomes and the strategies used to deal with them by HCWs and to identify the perceived stressors among HCWs and to compare the results among HCWs working in COVID-19 and non-COVID-19 wards. The Department of Obstetrics and Gynaecology at our institute is catering to emergencies most of the time. It would be interesting to examine the added stress of COVID-19 in managing obstetrical emergencies. This would help us to develop an institute-based protocol so as to facilitate resilience and stress relief among HCWs.

## Materials and methods

Study design 

This was a cross-sectional online questionnaire-based study.

Participants

All HCWs in the Department of Obstetrics and Gynaecology at our institute were included after obtaining consent. HCWs were defined as all the staff members directly involved in patient care, such as doctors and nurses, or those indirectly involved, such as attendants, helpers, laboratory technicians, or house-keeping staff. They were further divided into two groups:

Group 1: COVID-19 Caregivers

This group included staff members who were directly involved in COVID-19 care, such as those working in the screening/triage area, COVID-19 obstetrics ward, COVID-19 intensive care unit (obstetrics beds only), COVID-19 operation theatre (OT), and COVID-19 labour rooms.

Group 2: Other HCWs

This group included staff members who were not directly involved in COVID-19 care, such as those working in non-COVID-19 areas in the Department of Obstetrics and Gynaecology.

The HCWs were randomly allocated COVID-19 and non-COVID-19 rotational duties, which were compulsory for all. However, it was ensured as per the COVID-19 guidelines that high-risk groups (immunocompromised individuals, active TB patients, pregnant HCWs, etc.) were excluded from direct COVID-19 duties.

Inclusion criteria

1. Participants giving consent.

2. Participants not diagnosed with any psychiatric illnesses currently and in the past.

3. Participants not on any psychotropic medications.

4. Participants involved in the care of obstetrical patients.

5. Participants of group 1 must have completed at least one spell (14 days) of COVID-19 duty.

Exclusion criteria

1. Participants not giving consent.

2. Participants diagnosed with any psychiatric illnesses currently or in the past.

3. Participants on any psychotropic medications.

4. Participants involved in the care of non-obstetrical patients.

5. Participants having a language barrier (not well versed in Hindi/English).

Sample size

A time-bound cross-sectional research design was used for this study. All completely filled responses received from HCWs within 21 days of sending the questionnaire were included in the study, and groups were classified based on available responses.

Study tool

A semi-structured questionnaire with 66 questions under five sections was used. It took around 15 minutes to complete the survey. The first section contained nine questions regarding demographic details. The second section consisted of 21 questions from a free validated standardized tool: the Depression, Anxiety and Stress Scale - 21 Items (DASS-21) [7], to assess the psychological state of HCWs. The third section comprised seven questions from the validated Insomnia Severity Index [8,9] to assess any impact on sleep; the permission to use this method was obtained. The fourth section contained a single question with multiple choices to choose among the various possible stressors. The fifth section contained 28 questions from a validated, standardized Brief Coping Orientation to Problems Experienced (Brief COPE) inventory [10] to examine the coping strategies employed by HCWS in response to the COVID-19 pandemic.

Procedure

The questionnaire survey was done using the online web tool (Google Forms; Google LLC, Mountain View, CA). The link was circulated on official obstetrics and gynaecology WhatsApp (Facebook, Inc., Menlo Park, CA) groups of HCWs at the study institute and was kept open for 21 days. Three reminders at intervals of six days were sent out for filling up the same. The questionnaire began with a section on informed consent where the respondent was given the option to opt out of the survey at any time. Confidentiality and anonymity of the respondents were ensured. All completely filled responses received within 21 days were included in the study and analyzed. The results among COVID-19 caregivers and other HCWs were compared with respect to the magnitude and type of mental health outcomes, perceived stressors, and coping strategies being implemented on individual levels.

Data management

The data was imported to a Microsft Excel (Microsoft Corporation, Redmond, WA) spreadsheet, and the analysis was done using SPSS Statistics version 21.0 (IBM, Armonk, NY). Categorical variables were presented in numbers and percentages (%), and continuous variables were presented as means ± SD and median with interquartile ranges. The normality of data was tested with the Kolmogorov-Smirnov test. If the normality was rejected, then a non-parametric test was used. Statistical tests were applied as follows:

1. Quantitative variables were compared using the Mann-Whitney test/Kruskal-Wallis test (as the data sets were not normally distributed) between the groups.

2. Qualitative and categorical variables were compared using the chi-square test.

3. Binary logistic regression (stepwise backward LR) was used to determine the adjusted estimates.

A p-value of <0.05 was considered statistically significant.

## Results

A total of 184 HCWs responded to the survey and filled the questionnaire. The response rate was 71.8% (184/256).

Demographics

The demographic characteristics of the respondents are summarized in Table 1. Out of 184 respondents, 112 (60.9%) were COVID-19 caregivers, and 108 (58.70%) were females; 77 (41.8%) were nursing staff; 72 (39.1%) were doctors; 116 (63.0%) were young HCWs (<30 years) and 100 (54.3%) HCWs were living alone, i.e., away from their families. Compared to other HCWs, COVID-19 caregivers included significantly more young people aged <30 years (79.4% vs. 37.5%; p-value: <0.001), more married people (53.6% vs. 33.3%; p-value: 0.007); more HCWs living alone (53.6% vs. 33.3%; p-value: 0.007) and more nursing staff (61.6% vs. 11.1%; p-value: <0.001). Also, more females were working as COVID-19 caregivers, though the difference was not statistically significant (64.3% vs. 50%; p-value: 0.055) (Table 1). Among 112 COVID-19 caregivers, 35 (31.3%), 18 (16.1%), 16 (14.3%), 13 (11.6%), 10 (8.9%), and 20 (17.9%) were working in the COVID-19 ward, triage area and emergency, suspect ward, COVID-19 OT, COVID-19 ICU, and COVID-19 labour room respectively.

**Table 1:** Demographic profile of the healthcare workers (n=184) *Fischer's exact test COVID-19: coronavirus disease 2019

Variable	Total healthcare workers, n (%)	COVID-19 caregivers, n (%)	Other healthcare workers, n (%)	P-value (chi-square)
Overall	184 (100)	112 (60.9)	72 (39.1)	
Age (years)				
21-30	116 (63.04)	89 (79.4)	27 (37.5)	<0.001*
31-40	42 (22.83)	17 (15.2)	25 (34.7)	
41-50	13 (7.07)	4 (3.6)	9 (12.5)	
>50	13 (7.07)	2 (1.8)	11 (15.3)	
Gender				
Female	108 (58.70)	72 (64.3)	36 (50.0)	0.055
Male	76 (41.30)	40 (35.7)	36 (50.0)	
Marital status				
Married	100 (54.35)	60 (53.6)	24 (33.3)	0.007
Unmarried	84 (45.65)	52 (46.4)	48 (66.7)	
Living with family				
No	84 (45.65)	60 (53.6)	24 (33.3)	0.007
Yes	100 (54.35)	52 (46.4)	48 (66.7)	
Work/job profile				
Doctor	72 (39.1)	39 (34.8)	33 (45.8)	0.135
Nursing staff	77 (41.8)	69 (61.6)	8 (11.1)	<0.001
Others	35 (19.0)	4 (3.5)	31 (43.1)	<0.001
Comorbidities				
Yes	24 (13.0)	14 (12.5)	10 (13.9)	
No	158 (85.9)	98 (87.5)	60 (83.3)	0.729
Unknown/not answered	2 (1.1)	-	2 (2.8)	

Mental health outcomes

The mental health outcomes of the respondents are summarized in Table 2 and Table 3. Compared to other HCWs, COVID-19 caregivers had significantly higher prevalence of symptoms suggestive of depression (26.8% vs. 11.1%; p-value: 0.010), anxiety (36.6% vs. 16.7%; p-value: 0.004), stress (22.3% vs. 5.6%; p-value: 0.002), and insomnia (39.3% vs. 19.4%; p-value: 0.006). Also, all levels (mild, moderate, and severe) of depression, anxiety, and insomnia were significantly higher among COVID-19 caregivers (Table 2). Overall, sleep disorders were significantly higher among young HCWs (p-value: 0.049); females were significantly more stressed out (p-value: 0.007) and depression was significantly more among HCWs living with their families (p-value: 0.032) (Table 3).

**Table 2:** Prevalence and degree of depression, anxiety, stress, and insomnia among healthcare workers (n=184) *Fisher's exact test COVID-19: coronavirus disease 2019

		Overall, n (%), n=184	COVID-19 caregivers, n (%), n=112	Other healthcare workers, n (%), n=72	P-value (chi-square)		COVID-19 caregivers, n (%), n=112	Other healthcare workers, n (%), n=72	P-value (chi-square)
Depression	Absent	146 (79.3)	82 (73.2)	64 (88.9)	0.010	Absent	82 (73.2)	64 (88.9)	0.033*
	Present	38 (20.7)	30 (26.8)	8 (11.1)		Mild to moderate	22 (19.6)	7 (9.7)	
						Severe to extremely severe	8 (7.1)	1 (1.4)	
Anxiety	Absent	131 (71.2)	71 (63.4)	60 (83.3)	0.004	Absent	71 (63.4)	60 (83.3)	0.014
	Present	53 (28.8)	41 (36.6)	12 (16.7)		Mild to moderate	27 (24.1)	8 (11.1)	
						Severe to extremely severe	14 (12.5)	4 (5.6)	
Stress	Absent	155 (84.2)	87 (77.7)	68 (94.4)	0.002	Absent	87 (77.7)	68 (94.4)	0.003*
	Present	29 (15.8)	25 (22.3)	4 (5.6)		Mild to moderate	15 (13.4)	4 (5.6)	
						Severe to extremely severe	10 (8.9)	0 (0.0)	
Insomnia	Absent	126 (68.5)	68 (60.7)	58 (80.6)	0.006	Absent	68 (60.7)	58 (80.6)	0.017
	Present	58 (31.5)	44 (39.3)	14 (19.4)		Subthreshold	31 (27.7)	9 (12.5)	
						Moderate to severe clinical	13 (11.6)	5 (6.9)	

**Table 3:** Association of selected variables with depression, anxiety, stress, and insomnia (n=184) FE: Fisher's exact test

	Age (years) (n=184)				Gender (n=184)		Married (n=184)		Living with family (n=184)		Comorbidity (n=182)	
	21-30	31-40	41-50	>50	Male	Female	No	Yes	No	Yes	No	Yes
Stress												
Normal	98	33	12	12	69	86	67	88	72	83	135	18
Mild	6	3	1	0	4	6	6	4	5	5	7	3
Moderate	6	2	0	1	1	8	5	4	2	7	7	2
Severe	6	2	0	0	0	8	6	2	5	3	7	1
Extremely severe	0	2	0	0	2	0	0	2	0	2	2	0
P-value	0.582				0.007 (FE)		0.203 (FE)		0.359 (FE)		0.314 (FE)	
Anxiety												
Normal	79	28	12	12	61	70	55	76	63	68	111	18
Mild	10	5	0	0	5	10	7	8	6	9	15	0
Moderate	17	3	0	0	5	15	12	8	9	11	18	2
Severe	4	2	1	1	2	6	4	4	3	5	6	2
Extremely severe	6	4	0	0	3	5	6	4	3	7	8	2
P-value	0.348				0.241		0.502 (FE)		0.810 (FE)		0.329 (FE)	
Depression												
Normal	90	33	11	12	64	82	64	82	70	76	126	18
Mild	11	3	1	0	6	9	8	7	4	11	12	3
Moderate	11	2	1	0	3	11	8	6	9	5	13	1
Severe	1	1	0	1	0	3	1	2	1	2	2	1
Extremely severe	3	3	0	0	3	3	3	3	0	6	5	1
P-value	0.584				0.29		0.817 (FE)		0.032 (FE)		0.476 (FE)	
Insomnia												
Normal	77	24	12	13	59	67	58	68	59	67	108	16
Subthreshold	28	11	1	0	11	29	19	21	18	22	34	6
Moderate and severe clinical	11	7	0	0	6	12	7	11	7	11	16	2
P-value	0.049 (FE)				0.074		0.844 (FE)		0.821 (FE)		0.944 (FE)	

Perceived stressors

Compared to other HCWs, COVID-19 caregivers perceived the following stressors significantly: distancing from family and friends [79 (70.5%) vs. 35 (48.6%); p-value: 0.003], scarcity of workforce [36 (32.1%) vs. 10 (13.9%); p-value: 0.005] and dealing with non-cooperative patients [36 (32.1%) vs. 07 (9.7%); p-value: <0.001]. Other stressors like lack of support from staff and administration, family and personal problems, and ethical dilemmas due to multiple new guidelines and information coming in every day were also perceived more by COVID-19 caregivers though the difference was not significant. Furthermore, COVID-19 caregivers experienced multiple stressors (>4) more often as compared to other HCWs [52 (46.4%) vs. 22 (30.6%); p-value: 0.032] (Table 4).

**Table 4:** Perceived stressors felt by healthcare workers (n=184) COVID-19: coronavirus disease 2019; PPE: personal protective equipment

Stressor	Overall, n (%)	COVID-19 caregivers, n (%), n=112	Other healthcare workers, n (%), n=72	P-value (chi-square)
Distancing from family and friends	114 (62.0)	79 (70.5)	35 (48.6)	0.003
Fear of getting infected	94 (51.1)	53 (47.3)	41 (56.9)	0.203
Fear of transmitting the infection	91 (49.5)	57 (50.9)	34 (47.2)	0.627
Increased workplace pressure	68 (37.0)	43 (38.4)	25 (34.7)	0.615
Lack of support from staff, administration, and organizational structure	63 (34.2)	44 (39.3)	19 (26.4)	0.072
Family and personal problems	63 (34.2)	44 (39.3)	19 (26.4)	0.072
Ethical dilemmas in patient management due to multiple new guidelines and information coming in every day	57 (31.0)	40 (35.7)	17 (23.6)	0.083
Incidents of assaults on healthcare workers	49 (26.6)	32 (28.6)	17 (23.6)	0.458
Scarcity of workforce due to the formation of multiple teams	46 (25.0)	36 (32.1)	10 (13.9)	0.005
Dealing with non-cooperative patients	43 (23.4)	36 (32.1)	07 (9.7)	<0.001
Excessive media exposure	39 (21.2)	23 (20.5)	16 (22.2)	0.785
Stigma in society associated with COVID-19 care	37 (20.1)	23 (20.5)	14 (19.4)	0.857
Inadequate supply of PPE and lack of clarity regarding its use	35 (19.0)	26 (23.2)	09 (12.5)	0.710
Number of stressors perceived				
0-4		60 (53.6)	50 (69.4)	0.032
>4		52 (46.4)	22 (30.6)	

Coping strategies

COVID-19 caregivers had significantly higher avoidant coping scores than other HCWs (p-value: 0.006) and the difference was also statistically significant with respect to avoidant subscales: behavioral disengagement (p-value: 0.015), denial (p-value: 0.034), substance use (p-value: 0.007), and venting (p-value: 0.027). Although there was no significant difference in approach coping scores between the two groups, informational coping was significantly higher among the COVID-19 caregivers (p-value: 0.002). Also, humour approach scores were higher among COVID-19 caregivers (p-value: 0.036) (Table 5).

**Table 5:** Coping strategy scores among healthcare workers as per Brief COPE scale *Mann-Whitney U test/Kruskal-Wallis test SD: standard deviation; IQR: interquartile range; Brief COPE: Brief Coping Orientation to Problems Experienced

Coping type	COVID-19 caregivers, n (%), n=112		Other healthcare workers, n (%), n=72		P-value*
	Mean (SD)	Median (IQR)	Mean (SD)	Median (IQR)	
Approach type	27.3 (9.48)	28.0 (20-35)	26.1 (9.91)	27.0 (16.5-34)	0.437
Avoidant type	20.5 (6.86)	19.5 (15-26)	17.7 (5.40)	16.0 (14-21)	0.006
Approach subtype					
Acceptance	4.7 (1.97)	4.5 (3-6.5)	4.8 (2.04)	5.0 (3-6.5)	0.894
Active	4.9 (1.84)	5 (4-6)	4.8 (2.04)	5 (3-6)	0.829
Emotional	4.3 (1.85)	4 (3-6)	3.9 (1.69)	4 (2-5)	0.166
Informational	4.5 (1.92)	4 (3-6)	3.7 (1.89)	3 (2-5)	0.002
Planning	4.5 (1.90)	4 (3-6)	4.6 (2.07)	5 (2-6)	0.935
Positive reframing	4.4 (1.98)	4 (2-6)	4.3 (2.11)	4 (2-6)	0.939
Avoidant subtypes					
Behavioral disengagement	3.7 (1.70)	4 (2-5)	3.2 (1.45)	2 (2-4)	0.015
Denial	3.4 (1.77)	3 (2-5)	2.9 (1.50)	2 (2-4)	0.034
Self-blame	2.7 (1.48)	2 (2-3)	2.4 (0.93)	2 (2-2)	0.289
Self-distraction	4.4 (1.94)	3 (2-4)	4 (1.64)	3 (2-4)	0.142
Substance use	2.6 (1.39)	2 (2-2)	2.1 (0.54)	2 (2-2)	0.007
Venting	3.6 (1.60)	3 (2-5)	3.1 (1.44)	2 (2-4)	0.027
No coping					
Humour	2.4 (1.11)	2 (2-2)	2.6 (1.12)	2 (2-3)	0.036
Religion	4.4 (1.89)	4 (3-6)	4.3 (1.93)	4 (2-6)	0.579

Risk factor analysis for mental health outcomes

The results of binary logistic regression analysis, after adjusting for confounders, revealed that living with family and perceiving multiple stressors appeared to be associated with increased risk of anxiety [adjusted odds ratio (aOR): 2.761, 95% CI: 1.028-7.413, p-value: 0.044; aOR: 1.355, 95% CI: 1.173-1.565, p-value: 0.000 respectively]. Again, being a COVID-19 caregiver appeared to be a risk factor for stress (aOR: 1.84, 95% CI: 1.206-2.820, p-value: 0.005) while being married and being a doctor were associated with lower risk of stress (aOR: 3.949, 95% CI: 0.025-0.975, p-value: 0.047; aOR: 8.928, 95% CI: 0.961-0.992, p-value: 0.003 respectively). Furthermore, depression, stress, and anxiety appeared to be interrelated, each being a risk factor for others.

As far as coping strategies were concerned, avoidant coping was found to be associated with insomnia (aOR: 1.143, 95% CI: 1.026-1.273, p-value: 0.015) while approach coping was associated with depression and stress (aOR: 1.070, 95% CI: 1.010-1.134, p-value: 0.021; aOR: 5.104, 95% CI: 1.012-1.178, p-value: 0.024 respectively). Also, approach coping appeared to be less associated with anxiety (aOR: 0.940, 95% CI: 0.889-0.993, p-value: 0.028) (Table 6).

**Table 6:** Results of the binary logistic regression analysis to evaluate the risk factors for psychological impact outcomes COVID-19: coronavirus disease 2019; SE: standard error; aOR: adjusted odds ratio

Dependent variable	Independent variable	Beta (β) coefficient	SE	Exp(β)/aOR	95% confidence interval	P-value
Anxiety	Age	-0.656	0.384	0.519	0.245-1.100	0.087
	Gender (female/male)	0.774	0.515	2.168	0.789-5.954	0.133
	Living with family (yes/no)	1.016	0.504	2.761	1.028-7.413	0.044
	Comorbidities (yes/no)	-2.183	0.842	0.113	0.022-0.588	0.010
	Stress (present/absent)	2.417	0.723	11.216	2.718-46.273	0.001
	Depression (present/absent)	2.966	0.626	19.412	5.690-66.223	0.000
	Stressors (number)	0.304	0.074	1.355	1.173-1.565	0.000
	Coping approach type (score)	-0.062	0.028	0.940	0.889-0.993	0.028
Depression	Doctor (yes/no)	0.008	0.006	1.008	0.997-1.020	0.164
	Stress (present/absent)	2.292	0.660	9.890	2.710-36.091	0.001
	Stressors (number)	-0.144	0.082	0.866	0.738-1.017	0.079
	Coping approach type (score)	0.068	0.029	1.070	1.010-1.134	0.021
	Anxiety (present/absent)	2.852	0.602	17.324	5.320-56.418	0.000
Stress	Married (yes/no)	-1.866	0.939	3.949	0.025-0.975	0.047
	Living with family (yes/no)	1.239	0.942	1.731	0.545-21.890	0.188
	Doctor (yes/no)	-0.024	0.008	8.928	0.961-0.992	0.003
	COVID-19 healthcare worker (yes/no)	0.612	0.217	1.84	1.206-2.820	0.005
	Insomnia (present/absent)	1.288	0.759	2.881	0.819-16.052	0.090
	Coping approach type (score)	0.088	0.039	5.104	1.012-1.178	0.024
	Anxiety (present/absent)	2.395	0.747	10.292	2.539-47.410	0.001
	Depression (present/absent)	2.159	0.745	8.395	2.011-37.342	0.004
Insomnia	Age	-0.810	0.534	0.445	0.156-1.266	0.129
	Married (yes/no)	1.193	0.712	3.297	0.816-13.312	0.094
	Doctor (yes/no)	-0.012	0.007	0.988	0.975-1.002	0.093
	Stressors (number)	-0.189	0.108	0.827	0.670-1.022	0.079
	Coping avoidant type (score)	0.133	0.055	1.143	1.026-1.273	0.015
	Coping approach type (score)	-0.057	0.043	0.945	0.868-1.028	0.187
	Anxiety (present/absent)	1.333	0.767	3.793	0.844-17.045	0.082
	Stress (present/absent)	1.714	0.877	5.551	0.996-30.945	0.051

## Discussion

The response rate of our study was 71.8%, which in other studies varied from 30% to 94% [1-3,9-11]. Out of 184 respondents in our study, 60.9% were COVID-19 caregivers while it was just 41.5% in a study by Lai et al. [1]. Similar to many other published studies, our study had more young workers, more HCWs living alone, and more nursing staff. This may be attributed to the conscious administrative decision to exclude vulnerable people (older and those with significant comorbidities) from direct COVID-19 care [1,3,9-13].

A study assessing mental health outcomes during the COVID-19 pandemic among obstetricians and gynaecologists concluded that they experience significantly higher rates of both major depressive disorder and generalized anxiety disorder compared to the UK-nationwide estimates. The subgroup analysis showed that anxiety was more common among female doctors compared to males [4]. Uzun et al. also found poorer mental health in COVID-19 employees in the obstetrics and gynaecology department [5]. We found depression, anxiety, stress, and insomnia in 20.7%, 28.8%, 15.8%, and 31.5% respectively of total HCWs and in 26.8%, 36.6%, 22.3%, and 39.3% of COVID-19 caregivers respectively. Lai et al. found 58.5%, 51.6%, 40.7%, and 76.35% of COVID-19 HCWs positive for depression, anxiety, insomnia, and distress respectively, which is much higher than what we found in our study [1]. These higher percentages may be due to the fact that China was the first country to be hit by this pandemic and a lack of preparedness to deal with this unknown novel virus would have played a significant role in causing distress to HCWs. Chatterjee et al. found depression, anxiety, and stress in 34.9%, 39.5%, and 32.9% of doctors respectively in the early phase of the pandemic in India [14,15]. The percentage of same was lower in our study, which might be due to better preparedness at our institute: multiple training sessions, adequate availability of personal protective equipment (PPE), better lodging and dining facilities, and the government's decision to provide insurance to COVID-19 workers. On the contrary, Tan et al. reported a much lesser prevalence of depression (8.1%), anxiety (20.7%), and stress (6.4%) in their study from Singapore, which may reflect their extremely well-organized healthcare system, adequate preparations to deal with the pandemic, and timely information sharing along with other employee-centric measures to protect them from any health hazard [3].

In the pre-COVID-19 era also, many studies found a higher incidence of depression, stress, and burn-out among HCWs dealing with emergencies [16,17]. Ghetti et al. found that obstetrics and gynaecology residents are more prone to stress and burn-out, thereby affecting patient care. So, they were offered Balint training for 12 months, which enhanced their confidence by improving their psychological outlook and interest in patient care [18]. Thakrar et al. also observed poor mental health among HCWs in emergency departments [19]. A cross-sectional multicentre study conducted in eight cities in Iran on obstetrics and gynaecology specialists managing pregnant patients infected with COVID-19 showed significantly higher scores on a questionnaire evaluating depression as compared to other healthcare practitioners. Depression affected their quality of life too. This study also reported that social support improved some domains of quality of life like physical functioning, energy/fatigue, and emotional well-being [20].

We also found a significantly higher prevalence of all levels of depression, anxiety, and stress as well as insomnia among COVID-19 caregivers in our department, which is consistent with other studies [1,4,5,9-12,13,15]. From our findings, we presume that COVID-19 HCWs in other emergency departments are also highly susceptible to all psychiatric symptoms during the ongoing COVID 19 pandemic.

The adjusted analysis in our study showed that living with family appeared to be associated with increased risk of anxiety, which can be attributed to the associated apprehension of carrying the infection to home; while being married was associated with a lower risk of stress, which can be explained by the availability of support system to deal with stress. It has been suggested that remaining connected to family members through video calls acts as a major stress buster. Lack of family support and being unmarried have shown an association with depression, anxiety, and distress in studies from China. These studies also inferred that social support reduces anxiety and stress and improves sleep quality among COVID-19 HCWs [9-10,21]. Shah et al. found that the most significant stressor among obstetricians was associated with being up-to-date with rapidly changing guidelines, pathways, and protocols related to COVID-19 practice [4]. However, thanks to multiple training sessions at our institute, we did not face this problem. In other studies, the fear of transmitting infection and getting infected topped the list of stressors, followed by PPE shortage and isolation from family [1,2,9,22]. At our institute, thanks to the adequate supply of seemingly good-quality PPE, this was the least reported stressor experienced by the HCWs.

There are not many studies that have evaluated coping strategies among HCWs involved in COVID-19 care. Our study attempts to do so and we found that young HCWs (<30 years), nurses, and COVID-19 caregivers had significantly higher avoidant coping scores compared to non-COVID-19 HCWs. Emre Umucu et al. have demonstrated a positive association of COVID-19-related perceived stress with coping strategies: denial, substance use, behavioural disengagement, venting, religion, and self-blame. Our study revealed similar findings [21].

Studies have reported that the kind of coping style can determine the occurrence of psychological distress. Practising a negative coping style leads to substandard mental health with long-term consequences [23]. Koinis et al. reported that symptoms of stress and depression decrease with a positive approach while they increase with avoidant coping [24]. Eisenberg et al. have suggested that avoidant coping is not preferable to manage anxiety while approach coping results in devising better responses to deal with the adversity [25]. Another study suggests that employment of predominantly avoidant coping strategies leads to poorer quality of life and increased frequency of depression [26]. The number of perceived stressors and the use of avoidant coping strategies are positively correlated with all grades of depression, anxiety, and stress [27]. A prospective study by Holahan et al. including 1,211 middle-aged men and women reported that avoidant coping leads to both chronic and acute life stressors after four years and depressive symptoms after 10 years. In our study, avoidant coping was found to be associated with increased insomnia. Other studies on dementia caregivers and adolescents also endorse this finding [28-30].

Strengths of the study

1.This comprehensive evaluation of mental health was conducted in an already busy maternity ward with a very good response rate.

2. The coping mechanisms were also studied in detail so as to conduct workshops accordingly to help our dedicated COVID-19 staff in maintaining sound mental health.

3. The HCWs who screened positive for psychological issues were referred to the Department of Psychiatry for further management.

Limitations of the study

1. Only one institute was included in the study, resulting in a modest sample size and thereby limiting the generalizability of the findings.

2. This was a cross-sectional study, and we did not follow up on the findings.

## Conclusions

COVID-19 HCWs in the Department of Obstetrics and Gynaecology at our institute have particularly shown increased susceptibility to depression, anxiety, stress, and insomnia. They are primarily using avoidant coping mechanisms. It is possible that COVID-19 would have a long-lasting effect on the psychology of HCWs in already over-burdened maternity wards. 

Since the mental health of HCWs in India is already a neglected area, the evidence of additional stress of pandemic as revealed in our study warrants immediate interventions like reducing the stressors, providing psychological care, social support, and developing healthy coping mechanisms. At our institute, the Department of Psychiatry has initiated many counselling sessions where priority is being given to COVID-19 caregivers involved in emergency services.

## References

[1] Lai J, Ma S, Wang Y (2020). Factors associated with mental health outcomes among health care workers exposed to coronavirus disease 2019. JAMA Netw Open.

[2] Kang L, Li Y, Hu S (2020). The mental health of medical workers in Wuhan, China dealing with the 2019 novel coronavirus. Lancet Psychiatry.

[3] Tan BYQ, Chew NWS, Lee GKH (2020). Psychological impact of the COVID-19 pandemic on health care workers in Singapore. Ann Intern Med.

[4] Shah N, Raheem A, Sideris M, Velauthar L, Saeed F (2020). Mental health amongst obstetrics and gynaecology doctors during the COVID-19 pandemic: results of a UK-wide study. Eur J Obstet Gynecol Reprod Biol.

[5] Uzun ND, Tekin M, Sertel E, Tuncar A (2020). Psychological and social effects of COVID-19 pandemic on obstetrics and gynecology employees. J Surg Med.

[6] Wu P, Fang Y, Guan Z (2009). The psychological impact of the SARS epidemic on hospital employees in China: exposure, risk perception, and altruistic acceptance of risk. Can J Psychiatry.

[7] McMahon SA, Ho LS, Brown H, Miller L, Ansumana R, Kennedy CE (2016). Healthcare providers on the frontlines: a qualitative investigation of the social and emotional impact of delivering health services during Sierra Leone's Ebola epidemic. Health Policy Plan.

[8] Um DH, Kim JS, Lee HW, Lee SH (2017). Psychological effects on medical doctors from the Middle East Respiratory Syndrome (MERS) outbreak: a comparison of whether they worked at the MERS occurred hospital or not, and whether they participated in MERS diagnosis and treatment. J Korean Neuropsychiatr Assoc.

[9] Du J, Dong L, Wang T (2020). Psychological symptoms among frontline healthcare workers during COVID-19 outbreak in Wuhan. Gen Hosp Psychiatry.

[10] Xiao H, Zhang Y, Kong D, Li S, Yang N (2020). The effects of social support on sleep quality of medical staff treating patients with coronavirus disease 2019 (COVID-19) in January and February 2020 in China. Med Sci Monit.

[11] Huang JZ, Han MF, Luo TD, Ren AK, Zhou XP (2020). Mental health survey of medical staff in a tertiary infectious disease hospital for COVID-19 (Article in Chinese). Zhonghua Lao Dong Wei Sheng Zhi Ye Bing Za Zhi.

[12] Talevi D, Socci V, Carai M (2020). Mental health outcomes of the CoViD-19 pandemic. Riv Psichiatr.

[13] Thapa B, Gita S, Chatterjee K, Devrani A (2020). Impact of COVID-19 on the mental health of the society & HCW (healthcare workers): a systematic review. Int J Sci Healthc Res.

[14] Chatterjee SS, Bhattacharyya R, Bhattacharyya S, Gupta S, Das S, Banerjee BB (2020). Attitude, practice, behavior, and mental health impact of COVID-19 on doctors. Indian J Psychiatry.

[15] Kang L, Ma S, Chen M (2020). Impact on mental health and perceptions of psychological care among medical and nursing staff in Wuhan during the 2019 novel coronavirus disease outbreak: a cross-sectional study. Brain Behav Immun.

[16] Kumar S (2016). Burnout and doctors: prevalence, prevention and intervention. Healthcare (Basel).

[17] Khashaba E, El Sheref M, Ibrahim AAW, Neamatallah M (2011). Psychosocial stressors and hazards among emergency medical responders (EMR) in Mansoura City. Egypt J Occup Med.

[18] Ghetti C, Chang J, Gosman G (2009). Burnout, psychological skills, and empathy: Balint training in obstetrics and gynecology residents. J Grad Med Educ.

[19] Thakrar A, Raheem A, Chui K, Karam E, Wickramarachchi L, Chin K (2020). Trauma and orthopaedic team members' mental health during the COVID-19 pandemic: results of a UK survey. Bone Jt Open.

[20] Vafaei H, Roozmeh S, Hessami K (2020). Obstetrics healthcare providers' mental health and quality of life during COVID-19 pandemic: multicenter study from eight cities in Iran. Psychol Res Behav Manag.

[21] Umucu E, Lee B (2020). Examining the impact of COVID-19 on stress and coping strategies in individuals with disabilities and chronic conditions. Rehabil Psychol.

[22] Chen Q, Liang M, Li Y (2020). Mental health care for medical staff in China during the COVID-19 outbreak. Lancet Psychiatry.

[23] Wang H, Xia Q, Xiong Z (2020). The psychological distress and coping styles in the early stages of the 2019 coronavirus disease (COVID-19) epidemic in the general mainland Chinese population: a web-based survey. PLoS One.

[24] Koinis A, Giannou V, Drantaki V, Angelaina S, Stratou E, Saridi M (2015). The impact of healthcare workers job environment on their mental-emotional health. Coping strategies: the case of a local general hospital. Health Psychol Res.

[25] Eisenberg SA, Shen BJ, Schwarz ER, Mallon S (2012). Avoidant coping moderates the association between anxiety and patient-rated physical functioning in heart failure patients. J Behav Med.

[26] Myaskovsky L, Dew MA, Switzer GE (2003). Avoidant coping with health problems is related to poorer quality of life among lung transplant candidates. Prog Transplant.

[27] Main A, Zhou Q, Ma Y, Luecken LJ, Liu X (2011). Relations of SARS-related stressors and coping to Chinese college students' psychological adjustment during the 2003 Beijing SARS epidemic. J Couns Psychol.

[28] Holahan CJ, Moos RH, Holahan CK, Brennan PL, Schutte KK (2005). Stress generation, avoidance coping, and depressive symptoms: a 10-year model. J Consult Clin Psychol.

[29] Taylor BJ, Irish LA, Martire LM, Siegle GJ, Krafty RT, Schulz R, Hall MH (2015). Avoidant coping and poor sleep efficiency in dementia caregivers. Psychosom Med.

[30] Herman-Stabl MA, Stemmler M, Petersen AC (1995). Approach and avoidant coping: implications for adolescent mental health. J Youth Adolesc.

